# Identification of Novel Loci Precisely Modulating Pre-Harvest Sprouting Resistance and Red Color Components of the Seed Coat in *T. aestivum* L.

**DOI:** 10.3390/plants13101309

**Published:** 2024-05-09

**Authors:** Svetlana D. Afonnikova, Antonina A. Kiseleva, Anna V. Fedyaeva, Evgenii G. Komyshev, Vasily S. Koval, Dmitry A. Afonnikov, Elena A. Salina

**Affiliations:** 1 Institute of Cytology and Genetics, Siberian Branch of the Russian Academy of Sciences, 630090 Novosibirsk, Russia; 2 Faculty of Natural Sciences, Novosibirsk State University, 630090 Novosibirsk, Russia; 3 Kurchatov Genomics Center, Institute of Cytology and Genetics, Siberian Branch of the Russian Academy of Sciences, 630090 Novosibirsk, Russia

**Keywords:** winter wheat, red-grained wheat, seed coat color, pre-harvest sprouting, association analysis, image analysis, *Tamyb10*

## Abstract

The association between pre-harvest sprouting (PHS) and seed coat color has long been recognized. Red-grained wheats generally exhibit greater PHS resistance compared to white-grained wheat, although variability in PHS resistance exists within red-grained varieties. Here, we conducted a genome-wide association study on a panel consisting of red-grained wheat varieties, aimed at uncovering genes that modulate PHS resistance and red color components of seed coat using digital image processing. Twelve loci associated with PHS traits were identified, nine of which were described for the first time. Genetic loci marked by SNPs *AX-95172164* (chromosome 1B) and *AX-158544327* (chromosome 7D) explained approximately 25% of germination index variance, highlighting their value for breeding PHS-resistant varieties. The most promising candidate gene for PHS resistance was *TraesCS6B02G147900*, encoding a protein involved in aleurone layer morphogenesis. Twenty-six SNPs were significantly associated with grain color, independently of the known *Tamyb10* gene. Most of them were related to multiple color characteristics. Prioritization of genes within the revealed loci identified *TraesCS1D03G0758600* and *TraesCS7B03G1296800*, involved in the regulation of pigment biosynthesis and in controlling pigment accumulation. In conclusion, our study identifies new loci associated with grain color and germination index, providing insights into the genetic mechanisms underlying these traits.

## 1. Introduction

Pre-harvest sprouting (PHS) is the process of early germination of mature cereal grains in the ear occurring usually in high moisture conditions before harvest [1]. PHS launches a cascade of various metabolic processes in the grain, including the breakdown of starch and endosperm proteins to release energy [2]. As result, the grain loses nutrients, swells and its coat splits. The use of severely sprouted wheat in bakery results in sticky dough and bread with impaired crumb texture, high crumb stickiness, and a darker crust color [3,4]. Wheat that is severely sprouted in the field is less suitable for products for human consumption, and is often relegated to animal feed. This results in significant harvest losses, leading to reduced economic return for the producer [5,6,7]. Resistance to PHS plays a crucial role in ensuring the productivity and sustainability of wheat cultivation [8].

The process of early seed sprouting is highly complex and depends on various factors, including environmental conditions, seed dormancy, spike structure, awn shape, and more [9]. PHS is a quantitative trait controlled by multiple QTLs or genes, which are important for breeding PHS-resistant varieties. Currently, 66 MQTLs distributed across all 21 wheat chromosomes have been identified for PHS causal genes [10]. Some of them are well studied and closely related to the processes of metabolism and signaling of abscisic acid (ABA), a plant phytohormone controlling seed dormancy and germination [9,11,12], and most of them are associated with grain color and seed dormancy.

It is widely known that grains with a red coat are more resistant to PHS in comparison with white ones [13,14]. In cereals, grain color is determined by the composition of pigments such as anthocyanins, flavonoids, and carotenoids in outer layers of the seed coat [15,16,17]. For example, the presence of anthocyanins determines the purple and blue color of seeds. The presence of carotenoids may result in a yellowish color. The presence of flavonoids such as proanthocyanidins and phlobaphenes provides a reddish-brown or dark brown coloration of seeds [16]. Coat pigmentation is also related to the physiological characteristics of the seed. For instance, flavonoids can prevent and inhibit oxidative processes caused by various abiotic factors, such as drought, extreme temperatures, and salinity [18,19]. They have osmoregulatory properties, protecting plants under challenging environmental conditions [20,21].

There are a number of genes involved in both seed coat color and PHS control. For example, wheat grain redness is controlled by *R-1* genes located on chromosomes 3A, 3B, and 3D [22]. Plants with recessive homozygous and non-functional alleles (*R-A1a*, *R-B1a*, and *R-D1a*) have white grains. Plants with at least one of the three homoeologs dominant in *R-1b* alleles have a red grain color and resistance to PHS [14]. *R-1* genes encode MYB10 transcription factors, which are involved in flavonoid biosynthesis and the ABA signaling pathway [14,23]. *TaDFR-B* is another gene affecting PHS resistance and seed color by controlling anthocyanin synthesis [24].

A number of genes in wheat control seed dormancy and have an impact on PHS resistance regardless of the seed color. *TaMFT* on chromosome 3A (*Mother of Flowering Locus T*, also referred as *TaPHS1*) is a member of the *FT* gene family and plays a role in the transition to flowering. *TaMFT* is a positive regulator of ABA sensitivity. Higher expression of this gene positively correlates with seed dormancy [25]. *TaMKK3* located on chromosome 4A (*Mitogen Activated Kinase 3*) is responsible for the phosphorylation of proteins, leading to signal transduction pathways. It is assumed to be involved in the ABA signal transduction pathway. Variations in the gene sequence, which result in decreased kinase activity, significantly reduce the grain’s ability to germinate [26,27]. *TaVp1* is orthologous to the *ABI3* (*ABA Insensitive 3*) gene in *Arabidopsis thaliana*. It was shown that the expression level of *TaVp1* is directly associated with both seed dormancy and the embryo’s responsiveness to ABA in wheat [28].

The search for genes controlling PHS in a color-independent manner is of special interest. For example, developing PHS-resistant cultivars in white-grained wheats is advantageous due to their lighter flour color and higher flour yield [29,30]. Usually, this research is based on an analysis of bread wheat population with identical seed color type variable in PHS-related traits. Such an analysis allows us to identify the novel gene loci involved in PHS control [24,29,30].

Previously, using a population of winter wheat *Triticum aestivum* L., which includes red- and white-grained varieties, we demonstrated that the germination index of red-grained ones significantly varied and did not depend on the number of dominant alleles of *Tamyb10* [31]. In this study, we used a panel of winter-common wheat varieties with red grain color, exhibiting varying resistance to pre-harvest sprouting. Our objective was to investigate the relationship between variations in red color of seed coat estimated using the digital image analysis and PHS characteristics, as well as to identify novel loci and genes associated with these traits.

## 2. Results

### 2.1. Variability in the Grain Color Component and the PHS Traits

Grain color was described using four color spaces, RGB, HSV, Lab, YCrCb, each consisting of three parameters. Additionally, we had two types of parameters: the first type consisted of mean values of component intensities for seed pixels (e.g., RGB_mR); and the second type characterized the dominant seed colors, which included three dominant colors designated as 1, 2 and 3. For instance, RGB_dCR_1 represents the first dominant cluster of the R parameter in the RGB color space. The color parameters are described in Table S1 of Supplementary File 1. A more detailed explanation of color assessment is presented in Section 4.3. Descriptive statistics of the grain color traits for 159 common red-grained winter wheat genotypes are shown in Supplementary File 2. To estimate trait variability, we calculated the coefficient of variation for them. Its values vary from 0.0083 (for Lab_dCa_3 characteristic, a* component of the third dominant cluster) to 0.0885 (HSV_dCS_3 characteristic, saturation component of the third dominant cluster), and most are between 0.03 and 0.06. Interestingly, the lowest coefficients of variation (~0.01) were observed for traits related to seed redness, i.e., mean values for the a* component of the L*a*b* color space (Lab_ma), Cr component of the YCrCb color space (YCrCb_mCr), and related components for dominant color clusters (Supplementary File 2).

**Figure 1:**
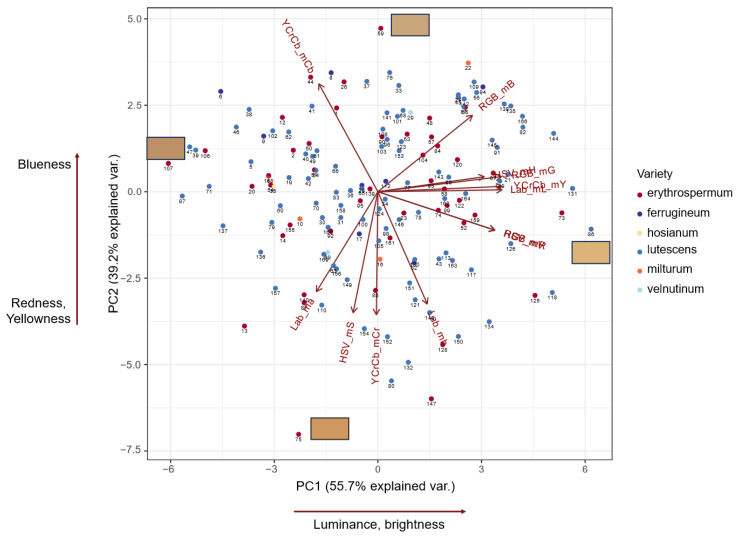
PCA biplot of grain color based on mean values of color parameters. Dots corresponding to wheat accessions are colored by their variety. Color bars represent grain color for accessions 59 (cv. ‘Victory’), 75 (cv. ‘Bruden’), 86 (cv. ‘Mv. Magdalena’), and 107 (cv. ‘AC Tempest’).

Figure 1 shows the principal component analysis (PCA) biplot for the distribution of accessions in the space, with 12 mean values for color components in four spaces. The analysis demonstrates that the total variation explained by two components is 95%. This plot demonstrates a positive correlation between lightness (HSV_mV, Lab_mL and YCrCb_mY variables) and PC1. Therefore, PC1 is related to seed color lightness. Seed color for accessions with high positive PC1 values is lighter; for accessions with negative PC1 values, it is darker. PC2 is correlated positively with seed blueness (RGB_mB, YCrCb_mCb) and negatively with redness (Lab_ma, YCrCb_mCr). It is also negatively correlated with Lab_mb (blue-to-yellow component) and HSV_mS (saturation). Seed color for accessions with high positive PC2 values is ‘colder’; for accessions with negative PC2 values, it is more reddish and ‘warmer’.

Figure 1 shows no remarkable clusters of accessions in the PCA diagram. The varieties are evenly distributed in this diagram, suggesting the absence of a relationship between variety and seed color in our population.

The distributions of most color traits (37 out of 48) exhibit a bell-shaped form close to Gaussian (Figure S1 in Supplementary File 1, Supplementary File 2; Shapiro–Wilk test *p* < 0.05). Most of the values in which the distribution deviates from normal are dominant cluster components. The distributions for 3 out of 12 traits for the mean values of the color components (Lab_mb, YCrCb_mCr, YCrCb_mCb) also deviate from the normal, likely due to asymmetry (see Figure S1 in Supplementary File 1).

To characterize the rate of pre-harvest sprouting susceptibility, the falling number (FN), germination index at the hard grain stage (GI_mat), and germination index at the late milk/hard dough stage (GI_milk) traits were evaluated. The accessions demonstrated variability in resistance to PHS ranging from high to low [31]. The falling number evaluated at the hard grain stage follows a normal distribution, with a mean value of 284 s (Figure S1 in Supplementary File 1). Some varieties demonstrate a large deviation from the mean FN, with values greater than 350 s and lower than 150 s, ranging from a minimum of 131 s to maximum of 425 s. These extreme values are typically considered characteristics of high and low susceptibility to PHS [32], while most accessions demonstrate medium PHS susceptibility.

The distribution of the germination index at the hard grain stage is bimodal with modes at 0 and 0.80; its minimum and maximum values are 0 and 0.98, respectively. This result demonstrates that the analyzed population has two groups of accessions with low and high GI_mat values [33]. The GI_milk parameter distribution is unimodal, asymmetric, and skewed towards lower values (skewness = 1.6). The majority of accessions (80%) have GI_milk values below 0.33, while some of them have higher values, demonstrating high susceptibility to PHS at this stage (Figure S1 in Supplementary File 1).

The analysis of Spearman’s correlation coefficients between 12 color and 3 PHS-related traits demonstrated no significant relationship at (*p* < 0.05) for most of the seed color/PHS trait pairs (Figure S1 in Supplementary File 1). Two exceptions are negative relationships between FN and RGB_mR, HSV_mV values (Spearman’s *r* = −0.19, *p* < 0.05). On the contrary, the correlation coefficients for most pairs of the color traits are highly significant (55 out of 66 pairs). This result is in agreement with the PCA analysis, demonstrating 95% of the variance explained by the first two components (Figure 1). The analysis also demonstrated a significant negative relationship between the FN and GI_milk values (Spearman’s *r* = −0.38, *p* < 0.05) and the absence of a significant correlation in the GI_mat/FN and GI_mat/GI_milk pairs.

Our analysis demonstrated the variability in the color characteristics in the population despite the fact that all the accessions have red seeds. The differences can be described by changes in the brightness of the seed (from dark to light) and the coloration (from red/warm to blue/cold). Our results also demonstrated a weak relationship between color and PHS-related traits.

We evaluated the heritability of PHS-related and seed color traits within the population. The results are presented in Table S2 (Supplementary File 1). The table demonstrates that the heritability of all PHS-related traits is high (*h*^2^ > 50%), suggesting a significant contribution of the genetic component to all these traits. For the color-related traits, all mean intensity values, except the RGB_mB (blue color component), have a heritability above 20%.

### 2.2. Relationship between Tamyb10 Dominant Allele Number and Red Color Components of Seed Coat

**Figure 2:**
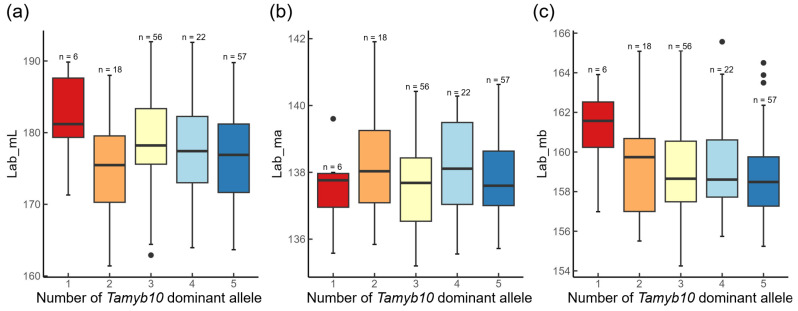
The boxplot of the distributions of the seed color traits (Y axis) depending on the *Tamyb10* number of dominant allele b (X axis). (**a**) Distribution of the lightness component, Lab_mL; (**b**) distribution of the Lab_ma component (green-to-red color); (**c**) distribution of the Lab_mb component (blue-to-yellow color).

We performed the characterization of 12 traits describing the mean color components of the four color spaces (as main color characteristics) in the winter red-grained wheat population to evaluate its possible relationship to *Tamyb10* allelic variation. The *Tamyb10* genotypes for each accession are provided in Supplementary File 3. The distribution of traits for Lab color space (Lab_mL, Lab_ma, Lab_mb) depending on the 5 *Tamyb10* allele b numbers is shown in Figure 2. We performed Welch ANOVA test for these and nine other traits and provide the results in Table S3 of Supplementary File 1. Our analysis indicated the absence of any statistical relationship between the *Tamyb10* genotypes and any seed color traits in the population.

### 2.3. Analysis of the Population Structure

Genotyping analysis yielded 24,145 SNP markers in total. After the removal of low-information sites (the fraction of missed SNPs > 0.2 and minor allele frequency (MAF) <0.05) and duplicates (three variants with identical positions and alleles), 19,863 variants remained. We applied an imputation procedure to these variants to obtain data for population structure description and association analysis (see Section 4).

**Figure 3:**
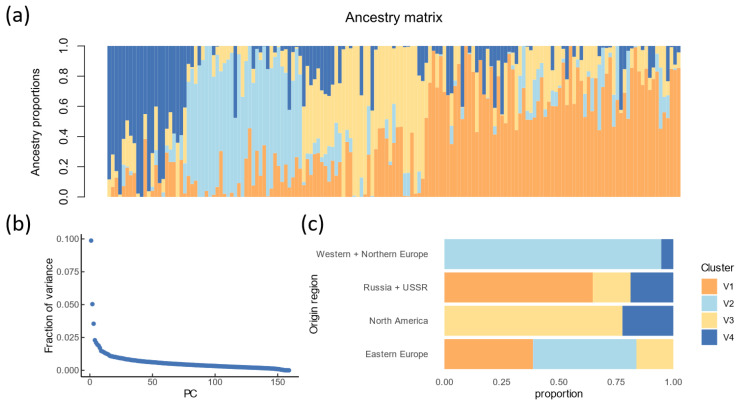
Analysis of the population structure. (**a**) Bar plot of admixture coefficients at *K* = 4. Colors represent ancestral clusters. Each bar designates individuals; all individuals are sorted by ancestry. (**b**) Fraction of variance explained by each principal component. (**c**) Distribution of ancestry clusters by geographic region from which varieties originate.

The result of the population structure analysis is shown in Figure 3A. The number of ancestral populations was estimated as *K* = 4 based on the PCA analysis of genetic variation: the first four principal components explained 10% of total variance (Figure 3B). The first ancestral population cluster includes the largest number of accessions, 43%. The second, third and fourth ancestral clusters contained 20%, 21% and 15% of accessions, respectively. The distribution of accessions from four dominant clusters by geographic regions is presented in Figure 3C. In general, the genetic composition is substantially linked to the geographical origin. Accessions from the first cluster originated from Russia/USSR and Eastern Europe regions (65% and 39%, respectively). Accessions from the second cluster originated from Western/Northern Europe and Eastern Europe (95% and 45%). Accessions from the third cluster originated mostly from North America (78%), Russia/USSR (16%) and Eastern Europe (16%). The fourth cluster comprised accessions originated from North America (22%), Russia/USSR (19%) and Western/Northern Europe (5%).

### 2.4. Association Analysis

We selected 38 markers by soft threshold *p* < 10^−4^ as a result of the whole-genome association analysis for all traits (see Supplementary File 4). Data for 13 markers selected by stringent criteria (*p* < 0.05 with correction by Benjamini–Hochberg method) are shown in Table 1.

**Figure 4:**
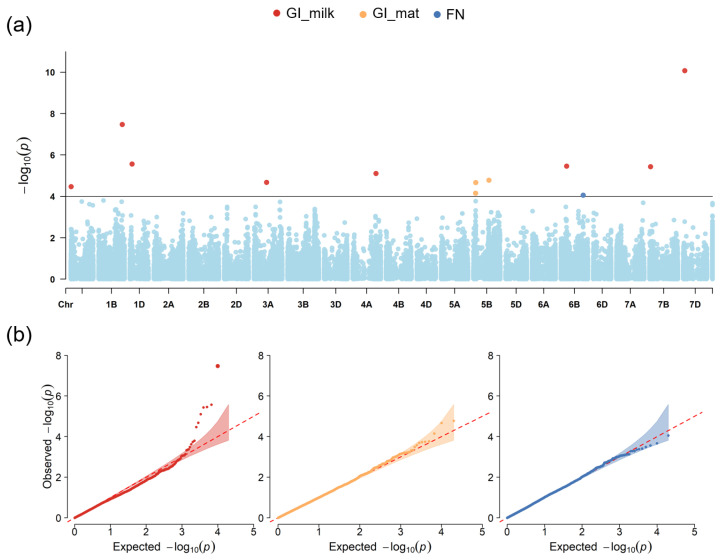
Results of genome-wide association analysis of the PHS-related traits. (**a**) Manhattan plot for GI_milk, GI_mat and FN characteristics. The correspondence between dot color showing the marker position and trait is above the plot. (**b**) Q-Q plots for each of the PHS-related traits. X-axis: expected −log_10_ (*p*) values. Y-axis: observed −log_10_ (*p*) values.

We observed 12 markers associated with PHS traits at *p* < 10^−4^ (Supplementary File 3). They were located on chromosomes 1A, 1B, 1D, 3A, 4A, 5B, 6B and 7D (Figure 4). Eight markers were associated with GI_milk, three markers on chromosome 5B were associated with GI_mat, and a single marker was identified for FN. Six markers associated with GI at the milk/hard dough stage were identified by applying stringent selection criteria (Table 1). Two SNPs on 1B and 7D explained up to 25.3% of phenotypic variance.

**Figure 5:**
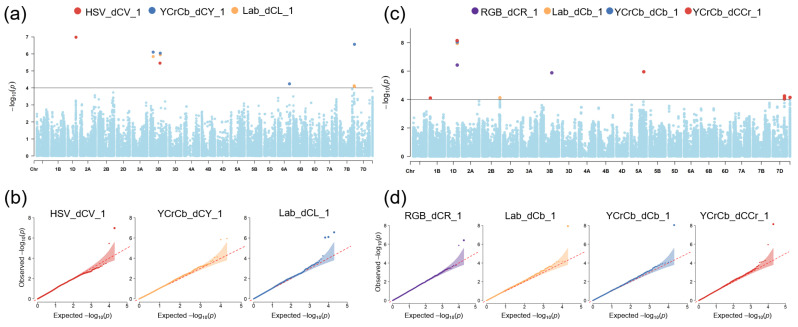
Results of genome-wide association analysis of the PHS-related traits. (**a**) Manhattan plot for lightness-related traits (HSV_dCV_1, Lab_dCL_1, YCrCb_dCY_1). The correspondence between dot color showing the marker position and trait is above the plot. (**b**) Q-Q plots for each of the lightness-related traits. (**c**) Manhattan plot for coloration-related traits (RGB_dCR_1, Lab_dCb_1, YCrCb_dCCb_1, YCrCb_dCCr_1). The correspondence between dot color showing the marker position and trait is above the plot. (**d**) Q-Q plots for each of the coloration-related traits. Axis description as in Figure 5.

We observed 26 markers associated with seed color traits at *p* < 10^−4^. They were located on chromosomes 1A, 1B, 1D, 2A, 2B, 3B, 5A, 6A and 7D (Figure 5 and Supplementary File 4). Five marker groups were identified on chromosomes 1D, 3B, 7B, 7D. Two SNPs on 3B chromosome were associated with three and more color traits (*Ku_c27771_508* and *RAC875_c35074_452*). SNPs on chromosome 1D were associated with brightness (HSV_V), and red and blue color components, spanning over more than 5 Mb and explaining from 23% up to 50% of the phenotypic variation. A group of multiple SNPs, as well as marker *Ku_c27771_508* on chromosome 3B, were associated with luminance and brightness parameters, as well as the red component of the RGB color space. Marker *GENE-3601_145* located on the 5A chromosome was significantly associated with one color trait only, YCrCb_dCCr_1, which is related to redness and explained 22.8% of variation. The marker group on chromosome 7B was linked to brightness characteristics and accounted for 13% of trait variation. SNPs localized on chromosome 7D, including marker TA015929-0626 linked to other two markers with an *r*^2^ > 0.9, were significantly associated with coloration parameters from both Lab and YCrCb color spaces.

Interestingly, our results demonstrate that loci associated with color traits did not overlap with loci associated with PHS-related traits. This implies that PHS and seed color are controlled by different genetic mechanisms in the red-grained wheat population. We also observe several markers associated with multiple traits for color characteristics but not for PHS-related traits. These results suggest that in red-grained wheat, some color traits may be controlled by the same genetic mechanisms, unlike PHS-related traits.

### 2.5. Gene Prioritization

Gene prioritization was performed for QTLs defined by the markers selected by stringent criteria (at corrected *p* < 0.05). According to the criteria, traits related to PHS were only represented by GI_milk (Table 1). For genomic regions around these markers, 101 genes were determined and used for gene prioritization, as described below (Supplementary File 5). Eight genes were differentially expressed in dormant/sprouting grains according to [34]. Among them, *TraesCS6B03G0368800* (RefSeq1.1 *TraesCS6B02G147200*) located on chromosome 6B was differentially expressed at a corrected false discovery rate (FDR) < 0.05 (Supplementary File 6).

The functions of 58 genes were found to be associated with PHS according to the KnetMiner database. Four of them are of particular interest because their expression patterns were similar to those of well-known genes involved in PHS (see Materials and Methods Section (Supplementary File 6)). These genes all have significant Spearman’s correlation coefficients for transcript per million (TPM) values in grain and some other tissues with at least one of the reference genes (Supplementary File 7). All of them are expressed in grain or related tissues. Two genes, *TraesCS6B03G0370300* (RefSeq1.1 *TraesCS6B02G147900*) and *TraesCS6B03G0374300* (RefSeq1.1 *TraesCS6B02G149500*), were located on chromosome 6B. The former encodes an aleurone layer morphogenesis protein with DSRM_SF domain (domain IDs PTHR33913, cd00048). The second gene encodes a protein containing plant calmodulin-binding domain (domain IDs IPR012417, PF07839). The third gene, *TraesCS4A03G0828000* (RefSeq1.1 *TraesCS4A02G333600*), is located on chromosome 4A. It encodes a protein containing CRAL/TRIO N-terminal domain (domain ID IPR011074) belonging to the CRAL-TRIO lipid binding domain superfamily. The fourth one, *TraesCS4A03G0826100* (RefSeq1.1 *TraesCS4A02G332600*), is also located on chromosome 4A. This gene encodes a protein containing F-box domain (domain ID IPR001810).

For QTL loci related to color traits, we identified 154 genes. Of them, 71 are located within *QGc.icg-1D* locus on the 1D chromosome, 48 from *QGc.icg-3B.1* and 11 from *QGc.icg-3B.2* on the 3B chromosome, 17 from *QGc.icg-5A* on chromosome 5A and 7 from the *QGc.icg-7B* on chromosome 7B (Supplementary File 5). A total of 102 genes were selected by their expression in the seed according to the expVIP database. Using KEGG resources, we were able to assign KEGG orthologous group identifiers to 34 genes. Only two of these genes were involved in pigment metabolism (Supplementary File 6). The gene located on chromosome 1D, *TraesCS1D03G0756600* (RefSeq1.1 *TraesCS1D02G318800*) with KEGG KO K00430, encodes a peroxidase participating in guaiacyl lignin, 5-Hydroxyguacyl lignin and syringyl lignin biosynthesis in the phenylpropanoid pathway. Another gene, *TraesCS7B03G1296800* (RefSeq1.1 *TraesCS7B02G482000*), is located on chromosome 7B and encodes 15-cis-phytoene synthase (PSY) involved in the carotenoid biosynthesis pathway.

The search for the KnetMiner database resulted in functional matches of 82 out of 102 genes related to color QTLs. A total of 7 of them have expression patterns similar to *Tamyb10*/*Tamyc1* genes. The list of prioritized candidate genes related to seed color is provided in Supplementary File 6. An analysis of their expression in grain and some other tissues is provided in Supplementary File 8. All of them except *TraesCS1D03G0758600* (RefSeq1.1 TraesCS1D02G319700) have significant Spearman’s correlation coefficients for TPM values in grain and some other tissues with at least one of the reference genes. *TraesCS1D03G0758600* is expressed in the grain at the soft dough stage. Two of these genes are of particular interest. Among them, a gene on the 1D chromosome, *TraesCS1D03G0758600* (RefSeq1.1 *TraesCS1D02G319700*), is involved in the flavonoid biosynthesis pathway. This gene encodes flavonol synthase (FLS), which participates in the synthesis of flavonols such as kaempferol and quercetin.

## 3. Discussion

### 3.1. Variability in the Red-Grained Winter Wheat Population in PHS and Seed Color Characteristics

The grain shell, comprising its permeability and color, is a fundamental factor influencing germination, a critical process during pre-harvest sprouting (PHS) [35]. Red-grained wheat varieties are generally recognized to exhibit greater resistance to pre-harvest sprouting compared to white-grained varieties [2]. However, even among red-grained varieties, pre-harvest sprouting can vary considerably [36].

In our study, we utilized a panel of red-grained winter wheat accessions exhibiting variations from high to low in resistance to PHS as determined by the analysis of germination index at the late milk/hard dough and mature grain stages, as well as the falling number. The heritability *h*^2^ of all PHS-related traits was estimated above 50%, suggesting the existence of certain genetic mechanism controlling these traits.

Grain color characteristics also exhibit variations despite the fact that all accessions have red-colored grains. These variations were detected by a digital phenotyping approach, allowing for the quantitative estimation of color parameters difficult to detect by naked eye [37]. The method estimates 48 parameters, which reflect various aspects of the seed color (hue, brightness, luminance, chroma intensity, and overall color uniformity), allowing for a detailed and precise color description [38]. Despite the high degree of correlation between seed color traits, we detected variation in the lightness characteristics and changes in chromaticity from redness to blueness. The heritability of grain color parameters varied; some of them had low heritability (less than 20%), while others displayed a larger genetic component (up to 50%), suggesting that the observed variation may be genetically controlled.

Previously, it has been assumed that the number of *Tamyb10* dominant alleles may influence both PHS resistance and grain color intensity and red color depth [22,39]. However, other investigations did not find any significant influence of the *Tamyb10* dominant allele number on sprouting [31,33,40]. Similarly, studies examining grain color using different methods on various sets of genotypes, including entirely red-grained varieties, also demonstrated the absence of a relationship between *Tamyb10* genes and variation in grain color for some genotype panels [36,41].

The relationship between PHS resistance and grain color in wheat is well known from many studies. For example, Rabieyan et al. demonstrated that the germination percentage in Iranian bread wheat has one of the highest correlation coefficients with color characteristics estimated from grain digital images such as L and b color components, brightness index and chroma [42]. Yan et al. demonstrated a significant correlation between the germination index and grain color (a/L ratio) in a set of 168 wheat varieties (lines) [43]. Yiwen et al. reported a significant relationship between PHS resistance and grain color in the population of 326 red- and white-grained cultivars [44]. Li et al. assessed solely PHS-related traits in the RILs population of red- and white-grained parents, and discovered some QTLs corresponding to *Tamyb10*, also known as a key regulator of both PHS resistance and seed color [45]. Interestingly, Zhu et al. described some markers detected by a 90K SNP array in a common wheat population of 192 varieties/lines which are not related to *Tamyb10* but influence both PHS resistance and grain color traits [46].

Here, we observed only a weak relationship between the estimated PHS-related traits and grain color parameters, mostly not statistically significant (Figure S1 in Supplementary File 1). Our genome-wide association study results demonstrated no overlap with the loci associated with PHS traits and seed color traits (Supplementary File 5). This is in agreement with the weak correlation between these traits. We also did not observe a relationship between the number of *Tamyb10* dominant alleles and seed color (Figure 2). Our results suggest that novel genetic mechanisms exist in the red-grained winter wheat population that control PHS resistance independently of color.

### 3.2. Comparing the Localization of Putative QTL with Existing Data

The genome-wide association study allowed us to identify novel loci and genes potentially associated with pre-harvest sprouting traits and grain color, aiming to propose molecular mechanisms for the regulation of these traits.

Markers associated with PHS were identified on chromosomes 1A, 1B, 1D, 3A, 4A, 5B, 6B, and 7D. Particularly, SNPs *AX-95172164* (chromosome 1B) and *AX-158544327* (chromosome 7D) explained a large proportion of the phenotypic variance (up to 25.3%), suggesting their potential utility in marker-assisted selection to enhance PHS resistance in wheat varieties.

Four of the detected loci overlapped with the previously identified loci controlling PHS. The *Qgi.icg-1D* locus within the 60-61.3 Mb interval on chromosome 1D (IWGSC RefSeq v2.1 assembly) is close to marker *wPt-665814,* found previously [36]. It is located at 64.6 Mbp (IWGSC RefSeq v2.1 assembly) and associated with spike sprouting and germination index [36]. Notably, this marker is also related to grain amylose content [47]. Another QTL, *Qgi.icg-4A*, is located within the 615.5–616.7 Mb on chromosome 4A (IWGSC RefSeq v2.1), close to *Qsd.sau-4A2*, located around 615.8 Mb [48]. Furthermore, marker *wsnp_BE497820B_Ta_2_1* on chromosome 5B at 414.8 Mb is proximal to the ABA 8’-hydroxylase *TaCYP707A2*/*TaABA8’OH2-5B* gene [49]. Using BLAST search, we determined the gene’s position on IWGSC RefSeq v2.1 between 420,431,488 bp and 420,435,720 bp. Another PHS-related gene, *TaQsd1*, is located at a distance of ~25 Mb from this marker. Using genome editing, Abe et al. reported *TaQsd1* to control seed dormancy, with triple gene knockout leading to a longer dormancy period [50]. BLAST search using *TaQsd1* sequences against IWGSC RefSeq v2.1 assembly showed that on chromosome 5B, this gene is located on ~390.7 Mb. The remaining loci we examined (see Supplementary File 5) are considered novel, as they were not identified in proximity to loci in previously published papers. They are located on chromosomes 1A, 1B, 3A, 5B, 6B, 7B, and 7D.

Other 26 significant SNP markers on chromosomes 1A, 1B, 1D, 2A, 2B, 3B, 5A, 6A, and 7D were associated with grain color traits. Notably, a QTL on chromosome 1D was linked to various grain color characteristics (brightness, red and blue parameters), explaining up to 50% of their variation. We hypothesize the presence of genes within this locus affecting grain color development. Our study’s grain color loci did not coincide with those identified by Arif et al., who employed the same seed color estimation method [51]. However, the locus containing the *TraesCS7B03G1296800* gene on chromosome 7B, encoding PSY, was previously associated with flour and endosperm color [52,53].

According to IWGSC RefSeq v2.1 assembly and annotation, *Tamyb10-A1* (~704 Mb), *Tamyb10-B1* (~773.2 Mb) and *Tamyb10-D1* (~572.2 Mb) are not within the QTLs we detected in the present study for neither PHS traits nor grain color.

### 3.3. Candidate Genes for Pre-Harvest Sprouting

We used gene prioritization with the aid of gene function, expression and metabolic networks’ databases and tools to identify potential candidates that may play a role in controlling pre-harvest sprouting (PHS) and color traits. Such approaches became very useful to pinpoint candidate genes for further detailed bioinformatics and experimental analysis [54,55,56,57].

The analysis of the expression patterns of genes known to be associated with PHS resistance revealed predominant expression in floral parts during flowering and in grains during their developmental stages [23,58,59]. Therefore, we applied one of the criteria for candidate gene selection, the similarity of expression pattern in various organs and stages of flower development, with known genes such as *TaMFT*, *Tamyb10* and *TaVp1*. We identified four genes located within QTLs that exhibited similar expression patterns (considered top candidates). The expression pattern of *TraesCS6B03G0370300* (Refseq1.1 *TraesCS6B02G147900)* is close to that of *Tamyb10* genes, particularly in anthers during flowering and in spikes at the early stages after pollination (Supplementary File 7). This gene encodes a DSRM_SF domain-containing protein involved in aleurone layer morphogenesis. Its homoeolog, *TraesCS6D02G109500* (RefSeq1.1 annotation), has been proposed as a candidate for moisture content (MC), a trait that typically determines grain storability. Its effect on moisture content is believed to influence water adsorption and loss properties by controlling the development of the aleurone layer, which constitutes the grain bran tissue, ultimately regulating water concentration in the grain [60]. Furthermore, the aleurone layer is a tissue where amylase and other hydrolytic enzymes are synthesized. These enzymes break down reserve materials in the starchy endosperm to nourish the growing seedling [61]. Therefore, the aleurone layer morphogenesis proteins may play a role in resistance to pre-harvest sprouting by controlling the structure and composition of the aleurone layer and influencing the enzymatic processes occurring in this tissue. To date, studies of homoelogs of this gene in other plants are lacking.

The second top candidate gene identified within the QTL on chromosome 6B is *TraesCS6B03G0374300* (RefSeq1.1 *TraesCS6B02G149500*), which encodes a calmodulin-binding domain-containing protein. However, there is limited information available about this gene and its homoelogs in other plant species, making it difficult to determine how it could be involved in PHS resistance.

The third of the top candidate genes, *TraesCS4A03G0828000* (RefSeq1.1 *TraesCS4A02G333600*), encodes a protein with a CRAL-TRIO lipid-binding domain. It is predominantly expressed in the spike and grain during the hard grain stage. *TraesCS4A02G333600* is orthologous to the *Oryza sativa* gene *Os01g0926800*, whose protein was annotated as a Sec14-like phosphatidylinositol transfer protein (SEC14L-PITP) [62]. Through a comprehensive investigation, it has been revealed that SEC14-like phosphatidylinositol transfer proteins (SEC14L-PITPs) across various plant species play a crucial role in determining membrane identity by facilitating the exchange of lipophilic substrates, thereby governing membrane signaling processes. Notably, plant SEC14L-PITPs typically exhibit a modular structure and are closely linked to essential cellular functions such as cell division, development, and stress responses [63]. Given these insights, it is conceivable that *TraesCS4A03G0828000* can be involved in sprouting by modulating signaling processes within wheat grain, where it is prominently expressed.

Finally, the *TraesCS4A03G0826100* gene (RefSeq1.1 *TraesCS4A02G332600*), located on chromosome 4A, encodes a plant F-box protein. Proteins of this ubiquitous F-box superfamily are involved in multiple processes in plants: stress response, plant development, and phytohormone signaling [64]. Other information about this gene or its homoelogs is insufficient to suggest any specific role in resistance to PHS.

### 3.4. Candidate Genes for Grain Color

In the current study, we identified genes most likely to be involved in grain color control in the red-grained wheat. Two of these genes are of particular interest. The first gene is *TraesCS1D03G0758600* (RefSeq1.1 *TraesCS1D02G319700*), encoding a flavonol synthase (FLS), located on chromosome 1D. The second candidate, *TraesCS7B03G1296800* (RefSeq1.1 *TraesCS7B02G482000*), encodes a phytoene synthase (PSY) and is located on chromosome 7B.

FLS is involved in the flavonoid biosynthesis pathway, where it promotes synthesis of flavonols (kaempferol, quercetin and isorhamnetin) from dihydroflavonols, which are synthesized from the flavonon naringenin by flavanone 3-hydroxylase (F3H) [65]. Dihydroflavonols can alternatively be utilized by dihydroflavonol 4-reductase (DFR), leading to the synthesis of anthocyanins and proanthocyanidins. This competition for substrate may result in an unbalanced accumulation of colorless flavonols or anthocyanins, conferring red, blue, and purple colors. This dynamic has been observed in multiple studies. Park et al. showed this in transgenic tobacco plants with the *O. sativa OsFLS* gene [66]. The transgenic plants exhibited paler and whiter petals and decreased anthocyanin compared to wild-type plants. Similarly, in *Arabidopsis thaliana*, overexpression of the *FLS1* gene resulted in altered seed color to light brown, and the seedlings possessed significantly less anthocyanin content compared to wild-type plants and *FLS1* mutants [67]. Conversely, *FLS1* mutants accumulated more anthocyanin than wild-type seedlings. Thus, in red-grained wheat, FLS could indirectly modulate anthocyanin content. A higher level of *FLS* expression presumably leads to lighter grain color, while a lower level of expression shifts biochemical reactions towards increased accumulation of colored pigments and grain color intensity.

Phytoene synthase PSY mediates the first step in the biosynthesis of carotenoids which confer yellow, orange and red colors [68,69]. Common wheat possesses three paralogs of phytoene synthase genes designated as *PSY1*, *PSY2* and *PSY3*, with three homoeologs each. The *TaPSY1* homoeologs are located on chromosome group 7, while the other two genes with their homoeologs are located on the fifth chromosome group [70]. *PSY1* was proposed to have a major effect on yellow pigment content in durum wheat, as its QTL on chromosome 7A was reported to explain up to 60% of yellowness variation and a homoeolog on chromosome 7B accounted for the majority of variation in endosperm color [53,71]. *PSY* is believed to be the rate-limiting enzyme controlling carotenoid flux and accumulation [72]. Indeed, in wheat, the down-regulation of PSY1 was observed in lines with decreased carotenoid accumulation in grains [73]. Hence, based on the results obtained, we propose that the *PSY1* gene contributes to the grain color of red-grained wheat by regulating carotenoid synthesis and accumulation.

## 4. Materials and Methods

### 4.1. Plant Material

Plant material included a winter common wheat (*Triticum aestivum* L.) collection provided by N. I. Vavilov All-Russian Institute of Plant Genetic Resources (VIR). This full collection consisted of 169 winter wheat accessions belonging to different varieties and included 16 breeding lines with different geographical origins. The collection was described previously [74]. Here, we analyzed 159 red-grained accessions only. The list of these accessions is provided in Supplementary File 3.

### 4.2. Evaluation of the Pre-Harvest Sprouting Traits

Two growing experiments were performed to evaluate pre-harvest sprouting (PHS) traits under different conditions: in field conditions in the Orel region (52°51′ N 36°26′ E) and in the greenhouse of the Center for Collective Use “Laboratory of Artificial Plant Growth” (FIC Institute of Cytology and Genetics, Novosibirsk). In the field experiment, seeds were sown in the autumn of 2021, and grains were harvested at the late milk/hard dough stage (GS77-GS87) [75] from the plants during the summer of 2022. For the greenhouse experiment, after a vernalization period of two months at +2 °C, five seedlings per accession were planted in bathtubs filled with ceramsite (expanded clay) as a substrate and subjected to a 16 h photoperiod with an average illuminance of 22 klx. Grains were collected at the hard grain (GS92-GS93) stages [75].

The falling number (FN) was evaluated for grains harvested in the Orel region in 2022 using the PCHP-7 (Biophysical Equipment, Russia) with the Hagberg–Perten method [76].

The germination index (GI) was evaluated at the milk/hard dough stage (GI_milk) and at the hard grain stage (GI_mat). Grains were placed on filter paper in Petri dishes with 10 mL of distilled water. There were three replicates with 24 grains each for each accession. The Petri dishes were placed in the climatic chamber (Weiss Technik, Balingen-Frommern, Germany,) for seven days at 22 °C and 50% humidity. Germinated grains were counted through daily observations. A grain was considered germinated when the coat above the embryo ruptured. At the end of the experiment, the viability of the remaining grains was assessed, and ungerminated grains were excluded from the GI calculation. The germination index was calculated using the equation described by Walker-Simmons [77]. As result, we obtained three traits describing seed susceptibility to PHS. A more detailed evaluation of pre-harvest sprouting traits was described in our recent work [31].

### 4.3. Grain Color Traits Estimation

To evaluate seed color traits, a method based on digital image analysis was implemented as described earlier [38]. For each accession, 15 grains were imaged in the laboratory conditions scattered on white A4 paper. ColorChecker color calibration card within the image area was used for color correction and image scale determination. Images were captured using a Canon EOS 600D digital camera equipped with a Canon EF 100 mm f/2.8 Macro USM lens. The digital image processing and color analysis was performed using SeedCounter application, version for desktop PC [37,38]. A single image per accession was used, since previous work demonstrated no significant differences between mean values of the seed traits obtained for several replicates [51]. Seeds were imaged before sowing in 2021.

To obtain seed color characteristics from images, four color spaces were used, RGB, HSV, Lab, YCrCb [78]. Each of them represents color as three components. The component values of one space can be obtained by transforming the component values of the other. The RGB color space is widely known and represents color in the intensities of the red (R), green (G) and blue (B) components of the pixel belonging to seed contour in the image. The advantage of using color representation in the HSV, L*a*b*, and YCrCb spaces is their ability to separate color into independent luminance/brightness/lightness (HSV V, L*a*b* L, YCrCb Y) and chromaticity components. This approach allows for a more reliable description of the coloration of a grain despite the differences in illumination of its parts due to its complex bulk shape.

The first type of color traits is mean values of component intensities for seed pixels. They represent the ‘average’ color of the seed in the image. The descriptors of the mean component values will be indicated by a lowercase m; for example, for the RGB color space, these are three parameters RGB_mR, RGB_mG, RGB_mB; for other spaces, the indications are similar.

The second types of color traits are dominant seed colors. To determine dominant colors, all seed pixels were grouped by color similarity into three clusters ranked by the number of pixels. In each of the three clusters, the values of three-color components for the centroid were determined. This procedure was performed for each color space. As a result, 36 traits of the second type were obtained. For example, for the RGB space, these are RGB_dC*j*_*i* parameters, where *j* = R,G,B is the color component designation, and *i* = 1,2,3 is the number of the dominant cluster. RGB_dCR_1 parameter is the R component for the first dominant color in RGB space. The use of three dominant colors allows for a more accurate estimation of uneven seed coloring.

In total, we considered 48 color characteristics for each seed. They are listed in Table S1 of Supplementary File 1. These characteristics were averaged for 15 seeds in the image to obtain seed color for each accession used for subsequent analysis.

### 4.4. Evaluation of the Relationship between Year and Seed Color Characteristics

The analysis of seeds from the plants of ITMI population demonstrated a linear relationship between some color traits and the harvesting year [38]. This relationship was attributed to a change in the pigment composition of the grains as a result of storage in the genbank. In the current work, grains were also represented by samples obtained in different years. A change in pigment composition depending on the storage time may lead to a systematic bias in the estimates of the color characteristics of the grains. To determine whether this factor was significant in our case, we used the JACOBI4 package [79] as described previously [38]. We subtracted the mean of the corresponding genotype from the values of the variables for each grain and evaluated Pearson’s correlation coefficient between every color trait and three variables encoding harvesting year (year; year rank; year class equal to 0 for 2013, 2014, 2015, 1 for 2016, 2017 and 2 for 2018, 2019). Two types of randomization tests, permutation and bootstrap with 2000 replicates, were used to evaluate the significance of the linear relationship between color trait and year variables. We obtained no significant linear relationship at (*p* < 0.05) for any of the seed color traits and year variables. Therefore, here, we neglected the bias from the linear relationship between harvesting year and color trait estimates.

### 4.5. Identification of Allelic Variations in the Tamyb10 Gene

The procedure of *Tamyb10* alleles analysis was previously carried out in the work of Fedyaeva et al. [31]. In summary, genomic DNA was isolated from the leaves of young plants collected from greenhouse according to the method described previously [80]. We used PCR markers developed by Himi et al. [81]. The distribution of *Tamyb10* alleles in the studied population of 159 red-grained accessions is presented in Supplementary File 3.

### 4.6. Statistical Analysis

A descriptive statistical analysis was performed with R 4.3, ggplot2, ggbiplot, dplyr and tidyr packages. To verify the normality of the trait distribution, the Shapiro–Wilk test was used. To test whether the trait values differ among replicates and genotypes, the Friedman test and one-way Welch ANOVA test were applied, respectively. Principal component analysis (PCA) was performed using R function prcomp(). Plots were obtained using the ggbiplot package. The psych package was used for the correlation analysis. To compare PHS traits means between different *Tamyb10* allelic dosage groups, the Kruskal–Wallis test was performed using the function kruskal.test().

### 4.7. Genotyping and Imputation

DNA isolation was performed using sodium bisulfite [82]. DNA was purified using a Bio-Silica Kit for DNA Purification following the manufacturer’s protocol. The concentration of the purified DNA was estimated with the Qubit dsDNA BR Assay (Thermo Fisher Scientific, Waltham, MA, USA) on a Qubit 4 Fluorometer (Thermo Fisher Scientific). Genotyping was implemented with Illumina Infinium 25K Wheat array by the TraitGenetics Section of SGS Institute Fresenius GmbH (Gatersleben, Germany, www.sgs-institut-fresenius.de, accessed on 1 August 2023). The SNPs with missing data above 20% and minor allele frequency (MAF) < 0.05 were excluded from further analysis using PLINK v. 2.0 [83]. The genotypes were imputed using BEAGLE v. 5.2 with default parameters [84].

### 4.8. Population Structure

The population structure was analyzed using the LEA R package [85]. To determine the optimal *K* ancestral populations, principal component analysis was performed. To provide formal evidence for a population structure, the Tracy–Widom test implemented in LEA was applied using the PCA eigenvalues.

### 4.9. Genome-Wide Association Analysis

The GAPIT R package [86] was used for genome-wide association analysis. Several models were tested for each of the trait (3 for PHS and 48 for seed coat color). The set of models included (1) a mixed linear model (MLM) with and without population structure and kinship (PCA.total = 10; Model.selection = TRUE; kinship.algorithm = “Vanraden”), (2) a compressed mixed linear model (CMLM) accounting for kinship and population structure, (3) a FarmCPU model and BLINK. The best model was selected based on quantile–quantile (Q-Q) plot analysis. The MLM model estimated narrow-sense heritability for each trait. Only those traits with heritability of more than 0.2 were considered for further analysis. For most of these traits, the best models were BLINK and FarmCPU; when both models were selected for the trait, we used the BLINK model. The CMLM model was determined to be the best for the population structure and kinship analysis. The Q-Q and Manhattan plots were created using the R package CMplot [87]. We selected a set of markers associated with the phenotypic traits by two criteria, soft (*p* < 10^−4^) and stringent (*p*< 0.05 with correction by the Benjamini–Hochberg method).

### 4.10. Genes Prioritization

Gene prioritization was performed for quantitative trait loci (QTLs) determined by markers that have a significant association with any of the traits analyzed using stringent selection criteria. These loci were determined by boundary markers separated by linkage disequilibrium (LD) distance. LD estimates were obtained by the Genetics R package [88]. LD decay plots were created with the R package LDheatmap [89]. For segregated markers, locus boundaries were determined by 500 Kb upstream and downstream of the marker coordinate. Genes within the QTLs were extracted using the IWGSC RefSeq v.2.1 annotation [90]. In the text, we referred to the prioritized genes by their IDs in the IWGSC RefSeq v.2.1 annotation, adding an ID from previous annotation version IWGSC RefSeq v.1.1 in parentheses.

For all genes within the QTL region, we performed a search of the relationship between their molecular functions and PHS processes/seed coat pigmentation. For genes from QTLs associated with PHS, gene expression data were considered first. We checked the presence of the gene ID within the list of differentially expressed genes in dormant and germinated seeds from the work of Zhang et al. [34]. Second, we compiled a list of *T. aestivum* genes associated with sprouting process from the KnetMiner database v. 5.7 [91] ranked by KnetScore (characteristic describing relevance of genes to the function terms). The list was obtained through the execution of the query ‘preharvest sprouting’. The expression patterns of the genes matching the query were analyzed using the Wheat Expression Browser powered by expVIP [92]. Genes exhibiting expression patterns most similar to those of well-studied PHS genes *TaMFT* (RefSeq1.1 *TraesCS3A02G006600*, *TraesCS3D02G004100*, *TraesCS3B02G007400*, *TraesCS3B02G010100*), *Tamyb10* (RefSeq1.1 *TraesCS3A02G472900*, *TraesCS3B02G515900*, *TraesCS3D02G468400*) and *TaVp1* (RefSeq1.1 *TraesCS3B02G452200*, *TraesCS3A02G417300*, *TraesCS3D02G412800*) were selected for detailed function analysis using the literature and the InterPro v. 98.0 protein domain database [93].

For the markers associated with seed coat color, we selected genes expressed in seeds first. For this purpose, we used data downloaded from the expVIP database, as described previously [51]. Genes were selected with TPM > 1 in seeds (“High level tissue” contains “seed”). Secondly, amino acid sequences for these genes were used as queries for KofamKOALA [94] and BlastKOALA [95] services to obtain their KEGG orthology group identifiers. Genes encoding proteins belonging to orthologous groups involved in pigment biosynthesis pathways listed in [51] were subjected to further examination. Third, we complemented the KEGG pathway analysis with the results obtained using KnetMiner platform. A list of genes obtained by the execution of the query ‘grain color’ was compared with the genes expressed in seeds determined at the first step of analysis. The selected genes were evaluated by their expression in the grain only or if they resembled the expression pattern of *Tamyb10* (RefSeq1.1 *TraesCS3A02G472900*, *TraesCS3B02G515900*, *TraesCS3D02G468400*) and *Tamyc1* (RefSeq1.1 *TraesCS2A02G409400*).

## 5. Conclusions

Resistance to pre-harvest sprouting is closely linked to grain coat characteristics, including its permeability and color, which are major factors affecting seed germination. In our study of a collection of red-grained common wheat varieties, we found that variation in coloration among these varieties does not correlate with resistance to pre-harvest sprouting. Consequently, when analyzing populations of wheat with red grain, we exclude grain color as a factor from our analysis. This exclusion enabled us to identify novel loci in the genome associated with pre-harvest sprouting and grain color and propose significant SNPs that could enhance wheat varieties during the breeding process.

Through the analysis of transcription data and gene annotation within the identified loci, we identified candidate genes conferring pre-harvest sprouting resistance, in addition to the known *Tamyb10* genes. Our analysis suggests that the structure and composition of the aleurone layer may play a role in this process by influencing enzymatic processes occurring within this tissue.

Furthermore, we hypothesize that variation in grain color characteristics, such as brightness, redness and blueness, is regulated by the balance between the synthesis of uncolored flavonols and colored anthocyanins, regulated by the activity of flavonol synthase. Another speculated mechanism underlying grain color is the carotenoid synthesis and accumulation pathway controlled by phytoene synthase.

## Figures and Tables

**Table 1:** Markers associated with PHS and grain color traits selected by stringent criteria. FN—Falling number; GI_milk—germination index at the late milk/hard dough stage (GS77-GS87). Color traits are described in Table S1 of Supplementary File 1.

Trait	Marker	Chr	Position RefSeq2, bp	*p*-Value	H&B Corrected *p*-Value	Allele	Effect	Explained Variance, %
PHS-related traits								
GI_milk	AX-95172164	1B	630,723,427	3.34 × 10^−8^	3.3 × 10^−4^	G	−0.09	24.1
	BS00094471_51	1D	60,703,757	2.70 × 10^−6^	1.5 × 10^−2^	G	−0.06	
	AX-94469815	4A	616,122,007	7.82 × 10^−6^	2.6 × 10^−2^	G	0.05	
	Kukri_c38732_246	6B	157,204,861	3.46 × 10^−6^	1.5 × 10^−2^	G	0.05	
	Kukri_c109962_396	7B	26,387,277	3.67 × 10^−6^	1.5 × 10^−2^	G	0.06	
	AX-158544327 ^1^	7D	40,278,555 ^1^	8.46 × 10^−11^	1.7 × 10^−6^	T	−0.12	25.3
Grain color traits								
YCrCb_dCCr_1	BS00075001_51	1D	416,729,513	7.08 × 10^−9^	1.4 × 10^−4^	G	1.07	31.7
RGB_dCR_1	wsnp_Ex_rep_c66423_64641115	1D	417,833,476	3.77 × 10^−7^	7.5 × 10^−3^	T	4.27	24.0
HSV_dCV_1	Excalibur_c48317_242	1D	418,514,802	1.05 × 10^−7^	2.1 × 10^−3^	T	−4.14	50.0
Lab_dCb_1		1D	418,514,802	1.15 × 10^−8^	2.3 × 10^−4^	T	−1.40	50.0
YCrCb_dCCb_1		1D	418,514,802	8.95 × 10^−9^	1.8 × 10^−4^	T	1.41	50.0
Lab_dCL_1	wsnp_Ex_c54357_57265797	3B	150,293,422	1.42 × 10^−6^	1.4 × 10^−2^	G	−2.31	16.2
YCrCb_dCY_1		3B	150,293,422	7.88 × 10^−7^	5.9 × 10^−3^	G	−2.28	16.9
HSV_dCV_1	Ku_c27771_508	3B	508,816,336	3.46 × 10^−6^	3.4 × 10^−2^	T	−4.23	
Lab_dCL_1		3B	508,816,336	1.12 × 10^−6^	1.4 × 10^−2^	T	−3.55	27.9
RGB_dCR_1		3B	508,816,336	1.31 × 10^−6^	1.3 × 10^−2^	T	−4.29	25.3
YCrCb_dCY_1		3B	508,816,336	8.97 × 10^−7^	5.9 × 10^−3^	T	−3.38	31.1
YCrCb_dCCr_1	GENE-3601_145	5A	582,358,406	1.11 × 10^−6^	1.1 × 10^−2^	T	−0.98	22.8
YCrCb_dCY_1	Excalibur_c81824_411	7B	752,467,841	2.72 × 10^−7^	5.4 × 10^−3^	T	−2.08	13.0

^1^ marker coordinates in RefSeq1.1 wheat genome annotation.

## Data Availability

The data are contained within this article.

## Supplementary Materials

The following supporting information can be downloaded at: https://www.mdpi.com/article/10.3390/plants13101309/s1, ‘Supplementary file 1.pdf’ (PDF format): Supplementary Tables S1–S3; Supplementary Figure S1. ‘Supplementary file 2.xlsx’ (Excel format): Descriptive statistics on the grain color traits of 159 common red-grained winter wheat genotypes. ‘Supplementary file 3.xlsx’ (Excel format): Sample list of 159 common red-grained winter wheat genotypes. ‘Supplementary file 4.xlsx’ (Excel format): SNPs associated with PHS traits and grain color. ‘Supplementary file 5.xlsx’ (Excel format): QTL boundaries and genes within them according to annotation. ‘Supplementary file 6.xlsx’ (Excel format): List of genes remained after the prioritization analysis. ‘Supplementary file 7.xlsx’ (Excel format): Co-expression analysis of the candidate genes related to PHS. ‘Supplementary file 8.xlsx’ (Excel format): Co-expression analysis of the candidate genes related to seed color.

## Abbreviations

ABA	Abscisic acid
cv.	Cultivar
FLS	Flavonol synthase
FN	Falling number
GI	Germination index
PCA	Principal component analysis
PHS	Pre-harvest sprouting
PSY	Phytoene synthase
QTL	Quantitative trait locus
SNP	Single-nucleotide polymorphism
TPM	Transcript per million
