# Supplementary material for: Identification of Novel Loci Precisely Modulating Pre-Harvest Sprouting Resistance and Red Color Components of the Seed Coat in T. aestivum L
Source: Plants (Basel). 2024 May 9;13(10):1309. doi: 10.3390/plants13101309 (PMC11126043; doi:10.3390/plants13101309)
Supplement: Supplementary file 1 [file plants-13-01309-s001.zip › Supplementary file1.pdf]

Supplementary file 1

for Afonnikova et al. paper "Identification of novel loci precisely modulating pre-harvest sprouting resistance and red color components of the seed coat in *T. aestivum* L.".

Supplementary Tables

Table S1. Color parameters and characteristics they define (see Afonnikov et al., 2021) for detailed description of the seed color traits.

Color space	Color parameter	Description
RGB[1]	RGB_mR[5]	Mean value of the Red component intensity for seed pixels
	RGB_mG[5]	Mean value of the Green component intensity for seed pixels
	RGB_mB[5]	Mean value of the Blue component intensity for seed pixels
	RGB_dCR_1, RGB_dCG_1, RGB_dCB_1[6]	R,G,B component values for the first dominant color cluster
	RGB_dCR_2, RGB_dCG_2, RGB_dCB_2[6]	R,G,B component values for the second dominant color cluster
	RGB_dCR_3, RGB_dCG_3, RGB_dCB_3[6]	R,G,B component values for the third dominant color cluster
HSV[2]	HSV_mH[5]	Mean value of the Hue component intensity for seed pixels
	HSV_mS[5]	Mean value of the Saturation component intensity for seed pixels
	HSV_mV[5]	Mean value of the Value/Brightness component intensity for seed pixels, HSV color space.
	HSV_dCH_1, HSV_dCS_1, HSV_dCV_1[6]	H,S,V component values for the first dominant color cluster
	HSV_dCH_2, HSV_dCS_2, HSV_dCV_2[6]	H,S,V component values for the second dominant color cluster
	HSV_dCH_3, HSV_dCS_3, HSV_dCV_3[6]	H,S,V component values for the third dominant color cluster
L*a*b*[3]	Lab_ma[5]	Mean value of the a* component intensity for seed pixels. These values are positive for red and negative for green color.
	Lab_mb[5]	Mean value of the b* component intensity for seed pixels. These values are positive for yellow and negative for blue color.
	Lab_mL[5]	Mean value of the Luminance component intensity for seed pixels.
	Lab_dCL_1, Lab_dCa_1, Lab_dCb_1[6]	L*,a*,b* component values for the first dominant color cluster

	Lab_dCL_2, Lab_dCa_2, Lab_dCb_2[6]	L*,a*,b* component values for the second dominant color cluster
	Lab_dCL_3, Lab_dCa_3, Lab_dCb_3[6]	L*,a*,b* component values for the third dominant color cluster
YCrCb[4]	YCrCb_mCr[5]	Mean value of the Cr component intensity for seed pixels. These values are positive and larger for red color.
	YCrCb_mCb[5]	Mean value of the Cb component intensity for seed pixels. These values are positive and larger for blue color.
	YCrCb_mY[5]	Mean value of the Luminance component intensity for seed pixels.
	YCrCb_dCY_1, YCrCb_dCCr_1, YCrCb_dCCb_1[6]	Y,Cr,Cb component values for the first dominant color cluster
	YCrCb_dCY_2, YCrCb_dCCr_2, YCrCb_dCCb_2[6]	Y,Cr,Cb component values for the second dominant color cluster
	YCrCb_dCY_3, YCrCb_dCCr_3, YCrCb_dCCb_3[6]	Y,Cr,Cb component values for the third dominant color cluster

[1] RGB color space represents color in the intensities of the red (R), green (G) and blue (B) components

[2] The HSV space represent color as combination of the hue (H, the similarity to red, yellow, green, and blue colors depending on the value), saturation (S, the higher saturation, the more pure is the color), and value/intensity (V, the higher the value, the brighter the color) components.

[3] CIE L*a*b* space expresses color as combination of the lightness (L*, the greater value, the lighter is the color) and color components a* (smaller values correspond to green, larger to red tones of the color) and b* (smaller values correspond to blue, larger values correspond to yellow tones).

[4] YCrCb space represent color as a combination of luminance or luma (Y, the greater value, the higher luminance), difference between the red component and luma (Cr, the greater value, the close color to red) and difference between the blue component and luma (Cb, the greater value, the close color to blue).

[5] Mean color components. To calculate them, the mean and standard deviations of intensities for each of the color component channels were estimated first, then the pixels, whose intensities differ from the mean by more than three standard deviations were excluded from the analysis. The mean value was calculated for the remaining pixels and used further.

[6] Dominant color clusters components. To determine dominant colors, all seed pixels were grouped by color similarity into three clusters. The clusters were ranked by the number of pixels they contained. In each of the three clusters, the values of the three color components for the centroid were determined. This procedure was performed for each color space and resulted, respectively, in nine color descriptors. For example, for RGB space, these are RGB_dC*j_i* parameters, where *j* is the color component designation, *i*=1,2,3 is the number of the dominant cluster. For example, RGB_dCR_1 parameter is the R component for the first dominant color in RGB space. The use of three dominant colors allows for a more accurate estimation of the uneven seed coloring.

All the color components values vary from 0 to 255, except components related to the H of the HSV space, whose values range was from 0 to 180.

Table S2. Heritability and best GWAS model of PHS and grain color traits. FN - Falling number; GI_milk - Germination index at the late milk/hard dough stage (GS77-GS87); GI_mat - Germination index at the hard grain stage (GS92-GS93); *_1 - the first dominant color; *_2 - the second dominant color; *_3 - the third dominant color; MLM2 - MLM with population structure and kinship.

Trait	Heritability, %	Best model
FN	56.8	BLINK; FarmCPU
GI_milk	52.8	FarmCPU
GI_mat	50.4	BLINK; FarmCPU
RGB_mR	46.2	BLINK; FarmCPU
RGB_mG	31.3	MLM2
RGB_mB	17.2	-
RGB_dCR_1	37	BLINK
RGB_dCG_1	26.7	MLM2
RGB_dCB_1	28.1	BLINK; FarmCPU
RGB_dCR_2	43.6	CMLM
RGB_dCG_2	25.8	BLINK; FarmCPU
RGB_dCB_2	6.9	-
RGB_dCR_3	11.9	-
RGB_dCG_3	0.2	-
RGB_dCB_3	0	-
HSV_mH	37.9	BLINK; FarmCPU
HSV_mS	26.5	BLINK; FarmCPU
HSV_mV	46.3	BLINK; FarmCPU
HSV_dCH_1	19.6	-
HSV_dCS_1	15.5	-
HSV_dCV_1	28	BLINK
HSV_dCH_2	35.7	CMLM
HSV_dCS_2	0.4	-
HSV_dCV_2	15.4	-
HSV_dCH_3	0	-
HSV_dCS_3	5.4	-
HSV_dCV_3	0	-
Lab_mL	34.8	BLINK; FarmCPU
Lab_ma	29.3	CMLM; MLM2
Lab_mb	45.9	CMLM; MLM2
Lab_dCL_1	34.5	BLINK
Lab_dCa_1	28.6	BLINK; FarmCPU
Lab_dCb_1	35.9	BLINK
Lab_dCL_2	28.9	BLINK; FarmCPU
Lab_dCa_2	16	-
Lab_dCb_2	42.5	CMLM; MLM2
Lab_dCL_3	0	-
Lab_dCa_3	2.8	-
Lab_dCb_3	24.5	BLINK; FarmCPU
YCrCb_mY	33.4	FarmCPU
YCrCb_mCr	35.6	BLINK; FarmCPU
YCrCb_mCb	48.9	CMLM; MLM2
YCrCb_dCY_1	31.9	BLINK

YCrCb_dCCr_1	37.4	BLINK
YCrCb_dCCb_1	36.1	BLINK
YCrCb_dCY_2	25.5	BLINK; FarmCPU
YCrCb_dCCr_2	33.3	MLM2
YCrCb_dCCb_2	44.8	CMLM; MLM2
YCrCb_dCY_3	0	-
YCrCb_dCCr_3	14.8	-
YCrCb_dCCb_3	22	BLINK; FarmCPU

Table S3. Comparing trait values between different *Tamyb10* dosage (1, 2, 3 and 5 dominant alleles). FN - Falling number; GI_mat - GI at hard grain stage; GI_milk - GI at hard milk/soft dough stage

**Trait**	**Test**	***p*-value**
RGB_mR	Welch's ANOVA	0.18
RGB_mG	Welch's ANOVA	0.16
RGB_mB	Welch's ANOVA	0.39
HSV_mH	Welch's ANOVA	0.12
HSV_mS	Welch's ANOVA	0.30
HSV_mV	Welch's ANOVA	0.18
Lab_mL	Welch's ANOVA	0.17
Lab_ma	Welch's ANOVA	0.30
Lab_mb	Welch's ANOVA	0.19
YCrCb_mY	Welch's ANOVA	0.17
YCrCb_mCr	Welch's ANOVA	0.31
YCrCb_mCb	Welch's ANOVA	0.19

Supplementary figure

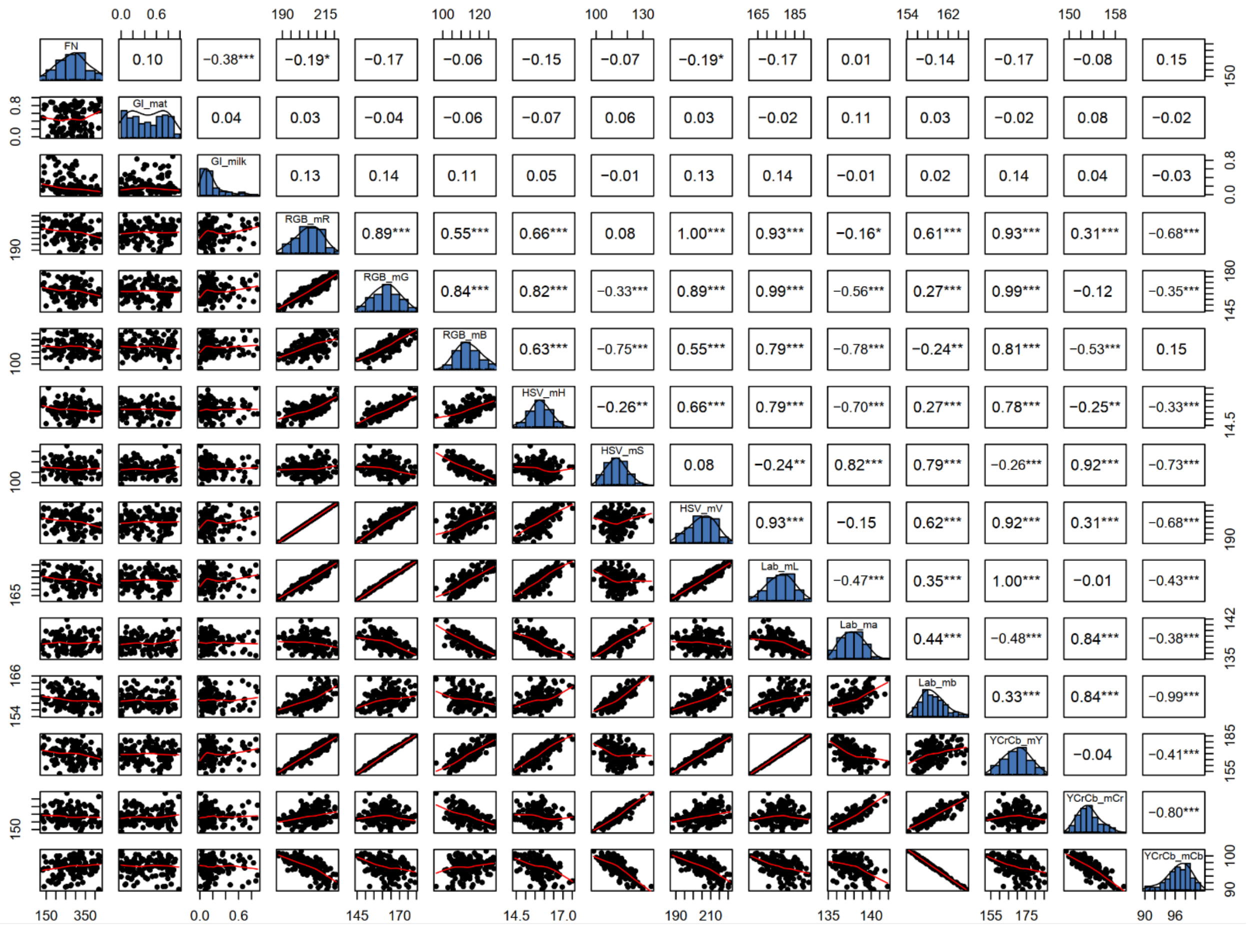

Figure S1. Distribution of the estimates of 15 seed traits in the population of red colored seed winter wheat and their pair-wise relationship statistics (Spearman's correlation coefficient).
